# Testicular Nuclear Receptor 4 Regulates Proliferation and Apoptosis of Bladder Cancer *via* Bcl-2

**DOI:** 10.3389/fmolb.2021.670409

**Published:** 2021-09-20

**Authors:** Huan Wang, Wenqin Luo, Xuliang Wang, Dingwei Xue, Liangliang Ren, Li Xu, Guangju Ge, Liqun Xia, Shicheng Yu, Mingchao Wang, Zhenwei Zhou, Gonghui Li, Haiyang Wu

**Affiliations:** ^1^ Department of Urology, Sir Run Run Shaw Hospital, Zhejiang University School of Medicine, Hangzhou, China; ^2^ Department of Urology, The Affiliated Hangzhou First People’s Hospital of Zhejiang University School of Medicine, Hangzhou, China

**Keywords:** testicular nuclear receptor 4, bladder cancer, apoptosis, proliferation, Bcl-2

## Abstract

Testicular nuclear receptor 4 (TR4) is a member of the nuclear hormone receptor family and acts as a ligand-activated transcription factor and functions in many biological processes, such as development, cellular differentiation, and homeostasis. Recent studies have shown that TR4 plays an important role in prostate cancer, renal cell carcinoma, and hepatocellular carcinoma; however, its potential link to bladder cancer (BC) remains unknown. This study found that bladder cancer exhibited a higher expression of TR4 compared to normal tissues. Overexpressed TR4 promoted the bladder cancer cell proliferation, and knocked down TR4 with TR4-siRNA suppressed the bladder cancer cell proliferation. Mechanistic studies reveal that TR4 functions by altering the expression of Bcl-2 to regulate apoptosis in bladder cancer cells. Furthermore, knocking down Bcl-2 reversed the BC proliferation induced by TR4. *In vivo*, we also confirmed that TR4 knockdown mice (TR4^+/−^) showed slower bladder cancer growth than wild-type mice (TR4^+/+^) induced by the carcinogenic chemicals. Moreover, TR4^+/−^ mice showed a lower grade of histopathology than the control group. In conclusion, these results indicate that TR4 plays a key role in bladder cancer proliferation, and targeting TR4 would probably be a potential strategy for bladder cancer treatment.

## Introduction

Bladder cancer (BC) is one of the most common cancers globally and brings a great burden on society (Antoni et al., 2017). BC accounted for nearly 3.0% of newly diagnosed cancer cases and 2.1% cancer-related deaths worldwide in 2018 (Bray et al., 2018). Moreover, the incidence of BC is expected to increase due to the growing and aging population (Chen et al., 2016; Richters et al., 2020). Significant effort has been made to develop an effective treatment method; however, patients with bladder cancer still have a poor prognosis, reflected in low 5-year survival rates, particularly in advanced stages (46% for stage 3 and 15% for stage 4) (Berdik, 2017). Therefore, there is an urgent need to explore the key paths of bladder cancer progression and identify promising targets for early diagnosis and treatment.

Testicular orphan receptor (TR4, also known as NR2C2) is a member of the nuclear receptor family. It was identified in 1994 by (Chang et al., 1994). Studies have indicated that TR4 acts as a transcriptional regulator and plays a significant role in diverse physiological processes, including neuronal and bone development Collins et al. (2004), Chen et al. (2005), fat metabolism, and fertility (Liu et al., 2007; Chen et al., 2008; Lin et al., 2017). Several studies regarding this receptor have been conducted. Therefore, its connection with cancers was uncovered and relevant mechanisms were explored. The effect of TR4 on urological tumors is one of the most studied topics. It has been reported that TR4 functions to suppress prostate tumorigenesis (Lin et al., 2014). Ding et al. found that the same nuclear receptor facilitated prostate cancer cell migration and invasion, accompanied by the upregulation of CCL2/CCR2 signals (Ding et al. (2015a), and possessed potential as a therapeutic target (Ding et al., 2015b). These inconsistent results might be due to the expression of another nuclear receptor, PPARγ (Lin et al., 2015a). In addition, suppression of TR4’s transactivation was considered a feasible solution to restore docetaxel sensitivity in castration-resistant prostate cancer (Hu et al., 2020). In renal cell carcinoma, TR4 was also spotted promoting vasculogenic mimicry formation and metastasis (Bai et al., 2018). Moreover, the knockdown of TR4 increases the sunitinib sensitivity in renal cell carcinoma (Shi et al., 2020). Investigations correlating with the diseases mentioned above are ongoing; however, little is known regarding TR4’s effect on bladder cancer initiation and progression.

As an antiapoptotic protein, Bcl-2 protein functions to downregulate mitochondrial outer membrane permeabilization (MOMP), subsequently blocking the outflow of proteins related to caspase activation (Kale et al., 2018). The effect of the Bcl-2 family on cancer development has been validated Delbridge et al. (2016), Montero and Letai (2018), and efficient targeted drugs (BH3 mimetics) have been certified by the FDA (Labi et al., 2008).

This study verified both *in vivo* and *in vitro* that TR4 regulates bladder cancer progression by promoting cancer cell proliferation. Furthermore, we confirmed that TR4 regulates the apoptosis by altering the Bcl-2 expression. These results imply that TR4 may serve as a target for the novel treatment of bladder cancer.

## Materials and Methods

### Clinical Specimens

BC tissue samples were obtained from patients who were diagnosed with BC and underwent surgery at Sir Run Run Shaw Hospital (Hangzhou, China). All patients’ clinical and follow-up information was complete and available between 2016 and 2019. All samples were collected with informed consent according to the Internal Review and the Ethics Board of Sir Run Run Shaw Hospital.

### Cell Culture

Human bladder cancer cell lines T24 and UM-UC-3 were obtained from the American Type Culture Collection (ATCC, Manassas, VA, United States) and were cultured in an RPMI-1640 medium containing 10% fetal bovine serum (Minhai Bio-Engineering, Lanzhou, China), and 100 IU/ml penicillin and streptomycin under 5% CO_2_ at 37°C. T24 and UM-UC-3 cell lines’ authentication was performed using short tandem repeat (STR) profiling within the last three years.

### Western Blot Analysis and Immunohistochemical Staining Analysis

Western blotting was performed as described in our previous study (Wang et al., 2020). Briefly, cells were collected and washed thrice with cold PBS. Then, total cellular proteins were extracted using the RIPA buffer with protease inhibitor cocktails (Millipore, Billerica, MA, United States) and quantified by bicinchoninic acid (BCA) analysis (Beyotime, China). Thereafter, the proteins were denatured at 100°C for 30 min. After that, proteins (10 μg) were added to the wells of 10% SDS–polyacrylamide gel and separated. Next, we transfected them onto PVDF membranes (Thermo Fisher Scientific, United States). After washing with TBST thrice and blocking in the skim milk on an incubator shaker for 2 h, the membranes were incubated with primary antibodies against GAPDH (1:1,000, sc-202525, Santa Cruz, CA, United States), TR4 (1:1,000, ab109301, Abcam, United States), and Bcl-2 (1:1,000, ER1706-47, Huabio, Hangzhou, China) at 4°C overnight, followed by 2-h incubation with IgG secondary antibody of mouse or rabbit against the primary antibodies. The proteins were detected using chemiluminescent detection reagents (FD8000, FUDE Biological Technology CO. ltd., Hangzhou, China).

Immunohistochemical (IHC) staining data were collected from the Human Protein Atlas (https://www.proteinatlas.org) (Uhlén et al., 2015; Thul et al., 2017). The expression of TR4 in different BC grades was calculated.

### siRNA and Plasmid Transfection

For siRNA transfection, T24 and UM-UC-3 cells (5×10^4^ per well) were seeded in a 6-well culture plate. When cells covered 40–50% surface of the plate, siTR4 (CGG​GAG​AAA​CCA​AGC​AA), siBcl-2 (GGA​UGA​CUG​AGU​ACC​UGA​A), or the negative control (purchased from Ribobio, Guangzhou, China) was transfected into the cells using the RFect siRNA/miRNA Transfection Reagent (Baidai, Changzhou, China) according to the manufacturer’s instructions. The cells were cultured in dishes for 48 h for subsequent evaluation.

For plasmid transfection, plasmids containing the TR4 or Bcl-2 cDNA full-length fragment designed and synthesized by GeneChem (Shanghai, China) were transfected into bladder cancer cell lines using a Lipofectamine 3000 kit (Invitrogen, Thermo Fisher Scientific, United States) according to the manufacturer’s instructions. The transfected cells were harvested for 48 h after transfection for further analysis.

### RNA Extraction and Quantitative Real-Time PCR

Total RNA was extracted from bladder cancer cells using the RNA-Quick Purification Kit (Yeasen Biotech, Shanghai, China), and the concentration of total RNA was measured using a Nanodrop 2000 (Thermo Fisher, United States). To synthesize cDNA required for quantitative real-time PCR, HiFiScript RT (CWBIO, Beijing, China) was used. After reverse transcription, samples (2 µl per well) were added to a mixture of 5-µl UltraSYBR Mixture (CWBIO, Beijing, China), 2-µl ddH_2_O, and 1-µl primers (0.5-µl forward primer+ 0.5 µl reverse primer) on a 96-/384-well plant. Quantitative real-time PCR was performed using a ROCHE PCR480 reaction apparatus (Roche Diagnostics, United States). The 2−▵▵Ct method was adopted to analyze the specific mRNA concentration. GAPDH served as the normalized control. The primers were synthesized by Tsingke Biological Technology (Beijing, China). TR4 primer sequence: forward: 5′‐GGC​TCT​GAA​CCT​GCC​TCT​G‐3′; reverse: 5′‐AGG​ATG​AAC​TGC​TGT​TTG​GG‐3′. GAPDH primer sequence: forward: 5′‐GGA​GTC​AAC​GGA​TTT​GGT‐3′; reverse: 5′‐GTG​ATG​GGA​TTT​CCA​TTG​AT‐3′; Bcl-2 primer sequence: forward: 5′-GGT​GGG​GTC​ATG​TGT​GTG​G‐3′; reverse: 5′-CGG​TTC​AGG​TAC​TCA​GTC​ATC​C‐3′.

### Cell Proliferation

The proliferation of bladder cancer cells was determined by both the CCK8 assay and EdU (5-Ethynyl-2′-deoxyuridine) assay. For the CCK8 assay, we planted cells resuspended in the medium into 96-well plates (100 µl per well) to ensure there were approximately 2,000 cells in each well. Cell viability was reflected by the absorbance of each well, measured at 450 nm with a microplate reader (Thermo Fisher, United States) after another 2-h incubation with a cell counting kit-8 reagent (Yeasen, Shanghai, China) under 5% CO_2_ at 37°C. The absorbance was quantified at 24, 48, and 72 h after cell seeding. For EdU assays, we used the EdU Cell Proliferation Kit (Meilunbio, Dalian, China) to evaluate cell proliferation. Newly synthesized DNA was labeled with red fluorescence and non-proliferative cells presented without red fluorescence, and the outcomes were observed and recorded using an inverted fluorescence microscope (Observer A1, ZEISS, Germany). Positive cells in three randomly chosen fields were quantified by normalizing to all Hoechst-stained cells.

### Cell Apoptosis

Cells seeded into 6-well plates were collected with their culture supernatant through tryptic digestion without EDTA, centrifuged, and resuspended in a binding buffer from an Annexin V-FITC/PI apoptosis kit (Multi Sciences, Hangzhou, China). Thereafter, 5-µl Annexin V-FITC and 10-µl PI were added to resuspend the mixture. After incubating for 5 min in the dark, flow cytometry analysis was performed.

### Protein–Protein Interaction Network

The online Search Tool for the Retrieval of Interacting Genes (STRING) database (http://www.string-db.org) was used to evaluate the interactions among different proteins (Szklarczyk et al., 2019). To discover other proteins interacting with TR4, we putTR4 into the STRING for further analysis. The interaction confidence score was set at 0.4.

### Histopathological Evaluation

Tumor tissues were fixed in 10% formalin and embedded into the paraffin. Paraffin-embedded blocks were cut into 5-μm-thick sections and stained with hematoxylin and eosin. Tumor progression was assessed based on the cellular morphology and depth of the tumor invasion.

### ChIP Assay

ChIP assays were performed using the SimpleChIP® Enzymatic Chromatin IP kit (CST, Massachusetts, United States) following the manufacturer’s instructions. Briefly, T24 cells (1×10^7^) were cross-linked with 1% formaldehyde for 10 min at room temperature. The ChIP dilution buffer was added, and the genomic DNA was sheared by sonication to an average size of 500 bp. After removing 2% of the solution for the evaluation of the input complex, the lysates were divided into two equal parts and immunoprecipitated overnight at 4°C using 5-μg TR4 antibody (ab109301, Abcam, United States) or IgG (CST, United States). The immunocomplexes were collected using 30‐μL salmon sperm DNA‐protein A agarose beads and washed sequentially with ChIP wash buffer 1, buffer 2, and buffer 3. DNA–protein complexes were collected in the ChIP elution buffer and disrupted by incubation at 65°C for 5 h in the presence of 312 mmol/L NaCl and 0.06-μg ribonuclease A/mL. Proteins were removed by digestion with proteinase K at 65°C for 2 h. The DNA from the input or immunoprecipitated samples was assayed by quantitative PCR (qPCR), and the products were identified by 2% agarose gel electrophoresis**.** Specific primers were designed to amplify a target sequence within the human Bcl-2 promoter, which are as follows: TR4RE1: F: 5′-TCA​GCC​CAA​CAA​GCC​AAC​TT-3′, R: 5′-TCA​TCC​CCT​CTT​GCC​TGG​ATA-3’; TR4RE2: F:5′-GGGTGCGCCATGAAAACAAG-3′, R:5′-TACACGGCTAGAAAGGGTCC-3’.

### *In vivo* Animal studies

Systemic TR4 knockdown mice (TR4^+/−^) with C57BL/6J were cultivated at the Nanjing Biomedical Research Institute of Nanjing University and confirmed by the Western blotting. Six- to eight-week-old mice with the same background (C57BL/6) were purchased from the SLAC (Shanghai Laboratory Animal Center) and were housed and fed under standard pathogen-free conditions. Thereafter, the mice were divided into two groups: TR4 knockdown mice (*n* = 5) and the control group (*n* = 5). After adding 0.05% N-butyl-N-(4-hydroxybutyl) nitrosamine (BBN) to the drinking water for a period of 20 weeks, the tumors were then measured and bladder tissues were taken for pathological examination to assess bladder urothelial hyperplasia and determine whether there was bladder intraepithelial neoplasia or tumor occurrence.

### Survival Analysis With TCGA Data

The TR4 expression data and clinical information of bladder cancer were obtained from the TCGA and GEO database (Bartha and Győrffy, 2021). A total of 200 patients with bladder cancer were enrolled in our research to analyze the relationship between the TR4 expression and bladder cancer prognosis. The overall survival and disease-free survival analyses were performed using the GEPIA2 (gepia2.cancer-pku.cn) (Tang et al., 2019).

### Statistics Analysis

Statistical analysis was performed using GraphPad Prism 8 (GraphPad Software, Inc. La Jolla, CA, United States) and SPSS (SPSS 24.0, Inc. United States). All data are presented as mean ± SD. Student’s t-test was used for comparison between the results of the two groups. *p* < 0.05 was considered statistically significant.

## Results

### TR4 Is Upregulated in Bladder Cancer and Associated With the Bladder Cancer Prognosis

**FIGURE 1:**
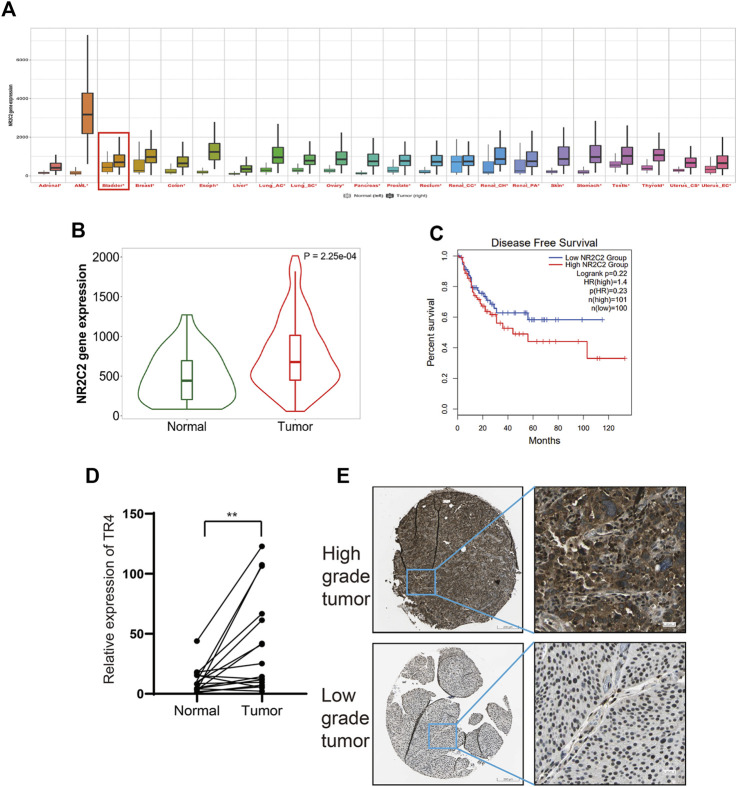
Correlation between TR4 and bladder cancer: **(A)** Expression of TR4 in pan-cancer, *p < 0.05. **(B)** Expression of TR4 in bladder cancer, *p* < 0.01. **(C)** Relationship between the disease-free survival and the expression of TR4 in bladder cancer, HR: hazard ratio. **(D)** mRNA level of TR4 in 16 tumor tissues and adjacent tissues, **p < 0.01. **(E)** IHC staining results of TR4 levels in high-grade bladder tumor and low-grade bladder tumor.

To study the role of TR4 in bladder cancer, we obtained the TR4 expression data from the TCGA and GEO datasets. We found that TR4 was upregulated in various cancers (Figure 1A). In bladder cancer, the TR4 expression was significantly higher in bladder cancer tissues (Figure 1B). Kaplan–Meier survival curves showed that TR4 expression was associated with the BC disease-free survival time and not with the overall survival time (Figure 1C and Supplementary Figure S1A). To verify the reliability of the public datasets, we examined the expression of TR4 in 16 bladder cancer tissues and adjacent tissues from Sir Run Run Shaw Hospital. We also found that the TR4 mRNA expression was higher in tumor tissues than in normal tissues (**p < 0.01, Figure 1D). Consistently, the results of IHC staining from the Human Protein Atlas database indicated that the TR4 expression was elevated in high-grade bladder cancer compared with low-grade bladder cancer (Figure 1E and Supplementary Figure S1B). These results indicate that TR4 possibly plays an important role in the prognosis of bladder cancer.

### TR4 Promoted the Bladder Cancer Proliferation

**FIGURE 2:**
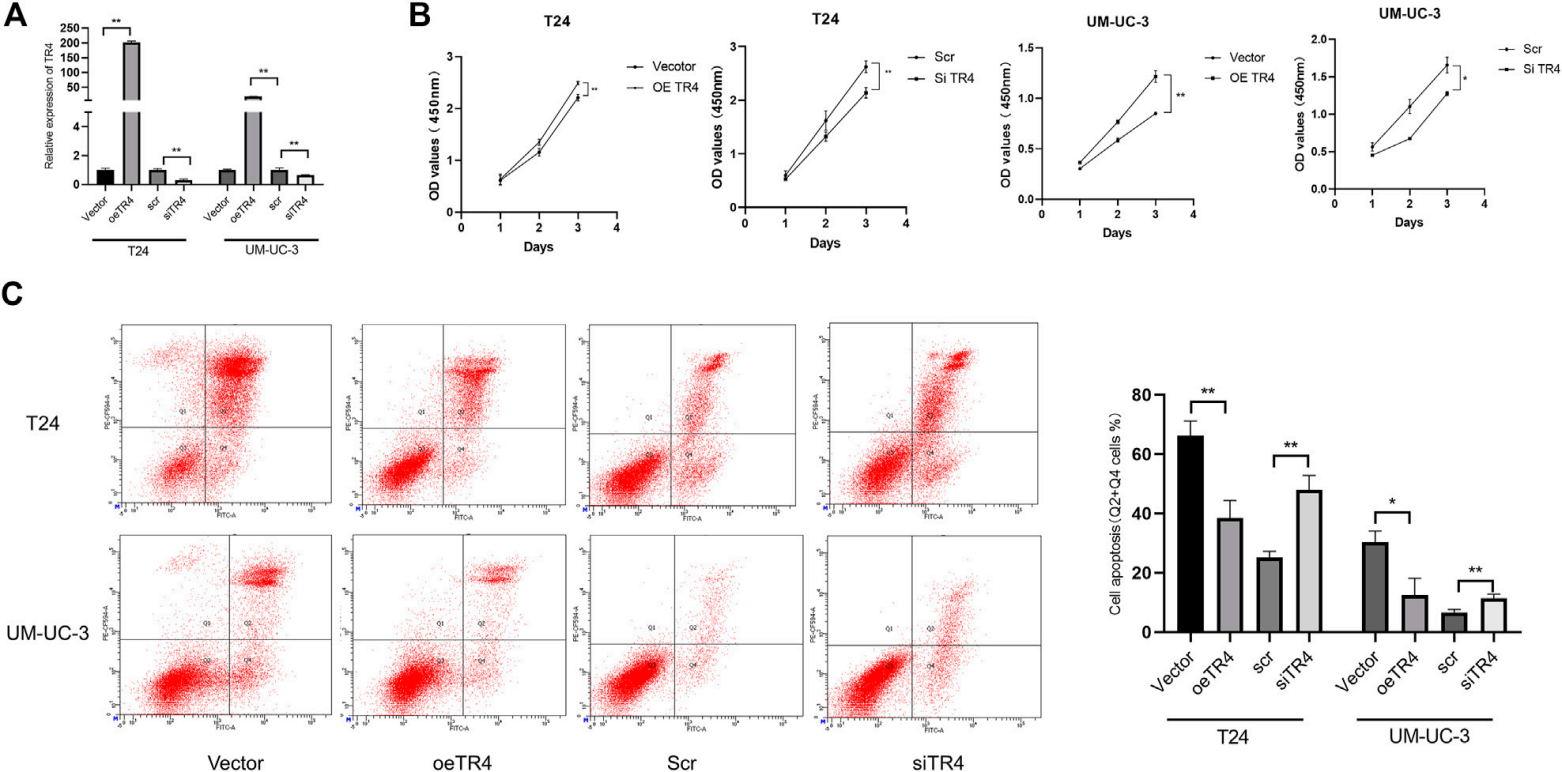
Effect of TR4 on proliferation and apoptosis in BC cell lines: **(A)** Relative TR4 mRNA expression was measured *via* qPCR after transfecting Vector, oeTR4, scr, and siTR4 in BC cell lines, including T24 and UM-UC-3. GAPDH was used as the internal control, **p < 0.05. **(B)** Vector, oeTR4, scr, and siTR4 were transfected to the T24 and UM-UC-3 cells. The relative cell viability was measured at 24, 48, and 72 h *via* the CCK8 assay, *p < 0.05, **p < 0.01. **(C)** Flow cytometry assay was used to evaluate the apoptosis analysis after transfecting Vector, oeTR4, scr, and siTR4, *p < 0.05, **p < 0.01.

**FIGURE 3:**
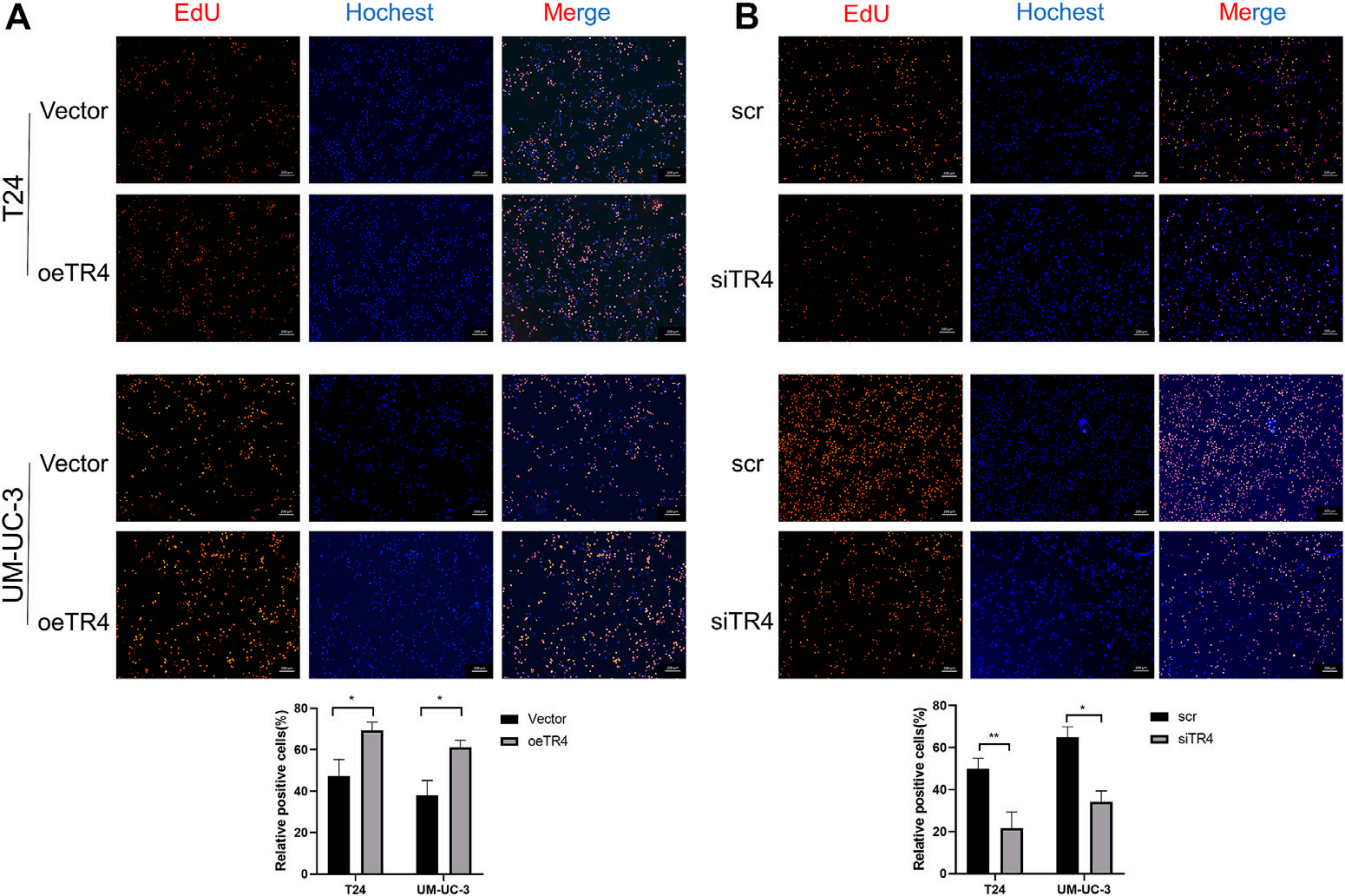
Effect of TR4 on viability in BC cell lines: **(A)** After transfecting Vector and TR4 in BC cell lines, the proliferation ability was measured by the EdU assay. Quantification of positive cells was determined by microscopy with ×40 magnification in three randomly chosen fields. Data are presented as mean ± SD. *p < 0.01. **(B)** After transfecting scr and siTR4 in BC cell lines, quantification of positive cells was determined in three randomly chosen fields. *p < 0.05, **p < 0.01.

To further investigate the function of TR4 in bladder cancer, we manipulated the TR4 expression by either the knockdown of TR4 with TR4-siRNA or overexpressing TR4 by adding functional TR4-cDNA, which was confirmed by qPCR (Figure 2A). We found that the overexpression of TR4 significantly promoted bladder cancer cell (T24 and UM-UC-3) proliferation using the CCK8 assay, whereas the knockdown TR4 expression inhibited the proliferation of BC cells (Figure 2B). Consistent with these results, the EdU assay also showed that tumor cell viability was associated with TR4 expression. In the T24 cell line, the overexpression of TR4 significantly increased the proliferation ability (Vector 47.43 ± 7.93% vs oeTR4 69.33 ± 4.04%, *p < 0.05, Figure 3A), while TR4 knockdown inhibited the proliferation ability (scr 50.22 ± 4.68% vs siTR4 21.67 ± 7.63%, *p* < 0.05, Figure 3B). Similar results were observed for UM-UC-3 cells. Taken together, these results suggest that TR4 promotes the proliferation of BC cell lines.

### TR4 Altered Cell Apoptosis of Bladder Cancer *via* Bcl-2

Furthermore, we also performed an apoptosis assay using Annexin V-FITC/PI double staining in both TR4 overexpressing and knockdown tumor cells. These results indicated that in T24 cell lines, the overexpression of TR4 significantly reduced the proportion of apoptotic cells from 66.27 ± 4.91% to 38.5 ± 5.957%, including the early and late apoptotic tumor cells (**p < 0.01). In contrast, TR4 knockdown significantly increased the percentage of apoptotic cells from 25.2 ± 2.007% to 47.93 ± 4.933% (**p < 0.01, Figure 2C). Similar results were observed in UM-UC-3 cells (Figure 2C).

**FIGURE 4:**
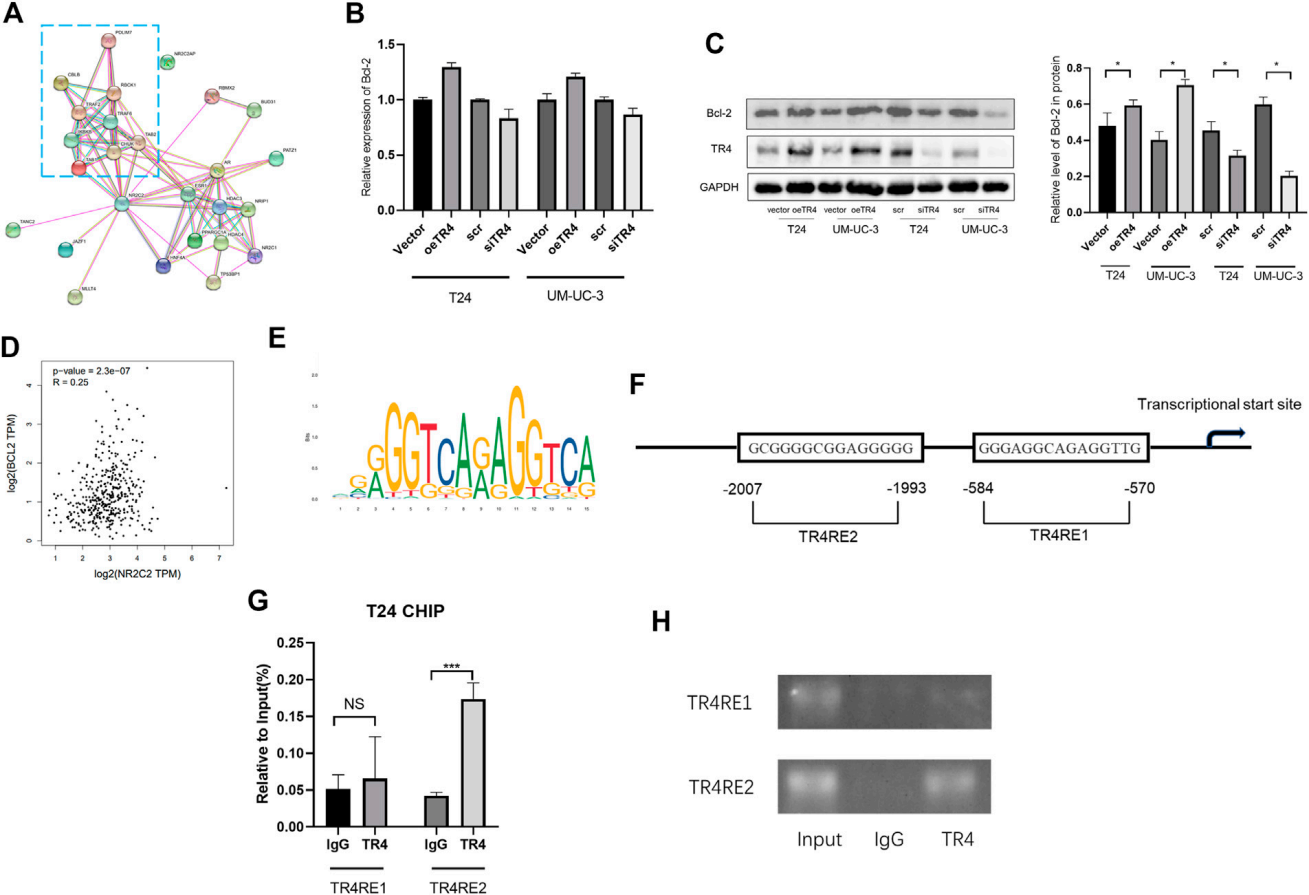
TR4 altered cell apoptosis of bladder cancer *via* Bcl-2: **(A)** The interaction network of TR4 was constructed, and the most relevant proteins involved in the cell apoptosis pathway were enclosed in a blue rectangle. **(B)** Bcl-2 mRNA expression was verified by qPCR after transfecting Vector, oeTR4, scr, and siTR4. **(C)** Bcl-2 protein expression was verified by Western blot after transfecting the Vector, oeTR4, scr, and siTR4. GAPDH was used as the internal reference. Semiquantification of Bcl-2 based on the band density from three independent experiments was shown in the right panel, *p < 0.05. **(D)** GEPIA was used to analyze the co-expression of TR4 and Bcl-2 in bladder cancer. Spearman = 0.25, *p* < 0.01. **(E)** TR4 motif sequence was predicted by Jaspar. **(F)** Bcl-2 promoter regions contain potential TR4 binding sites (TR4RE1 and TR4RE2). **(G)** and **(H)** ChIP assay results showed that TR4 binds to TR4RE2, NS: no significant, ***p < 0.001.

To analyze the potential interaction with TR4, a PPI network was constructed by using STRING. The PPI network contained the most relevant 25 proteins, including TNF receptor-associated factor 2 (TRAF2), TNF receptor-associated factor 6 (TRAF6), mitogen-activated protein kinase kinase kinase 7 (MAP3K7), TGF-beta activated kinase 1 binding protein 1 (TAB1), TGF-beta activated kinase 1 binding protein 1 (TAB2), conserved helix-loop-helix ubiquitous kinase (CHUK), receptor-interacting serine/threonine-protein kinase 1 (RIPK1), inhibitor of nuclear factor kappa B kinase subunit beta (IKBKB), and inhibitor of nuclear factor-kappa B kinase regulatory subunit gamma (IKBKG), which are involved in the NF-κB signaling pathway and death receptor signaling (Figure 4A). Bcl-2 family proteins, which are downstream proteins of NF-κB pathway, play a key role in the cell death or apoptosis. Thus, we hypothesized that Bcl-2 is a downstream protein of TR4. We observed that Bcl-2 was upregulated when TR4 was overexpressed at both the mRNA level and protein level. In contrast, TR4 knockdown significantly reduced the Bcl-2 expression (Figure 4B and Figure 4C). To determine the relationship between TR4 and Bcl-2, we conducted Spearman correlation analysis using TCGA (Figure 4D). It was clear that the expression of TR4 had a significantly positive correlation with Bcl-2 (Spearman = 0.25, *p* < 0.01). Previous studies have revealed that Bcl-2 is regulated by TR4 through the transcription (). Thereafter, the JASPAR database was used to search for potential TR4 response elements (TR4RE) in the promoter region of Bcl-2 (Figure 4E and Figure 4F). Subsequently, ChIP assays were performed and demonstrated that TR4 could bind to the Bcl-2 promoter region TR4RE2 (−1993 bp to −2007 bp), except TR4E1 (−570 bp to −584 bp) in T24 cells (Figure 4G and Figure 4H).

**FIGURE 5:**
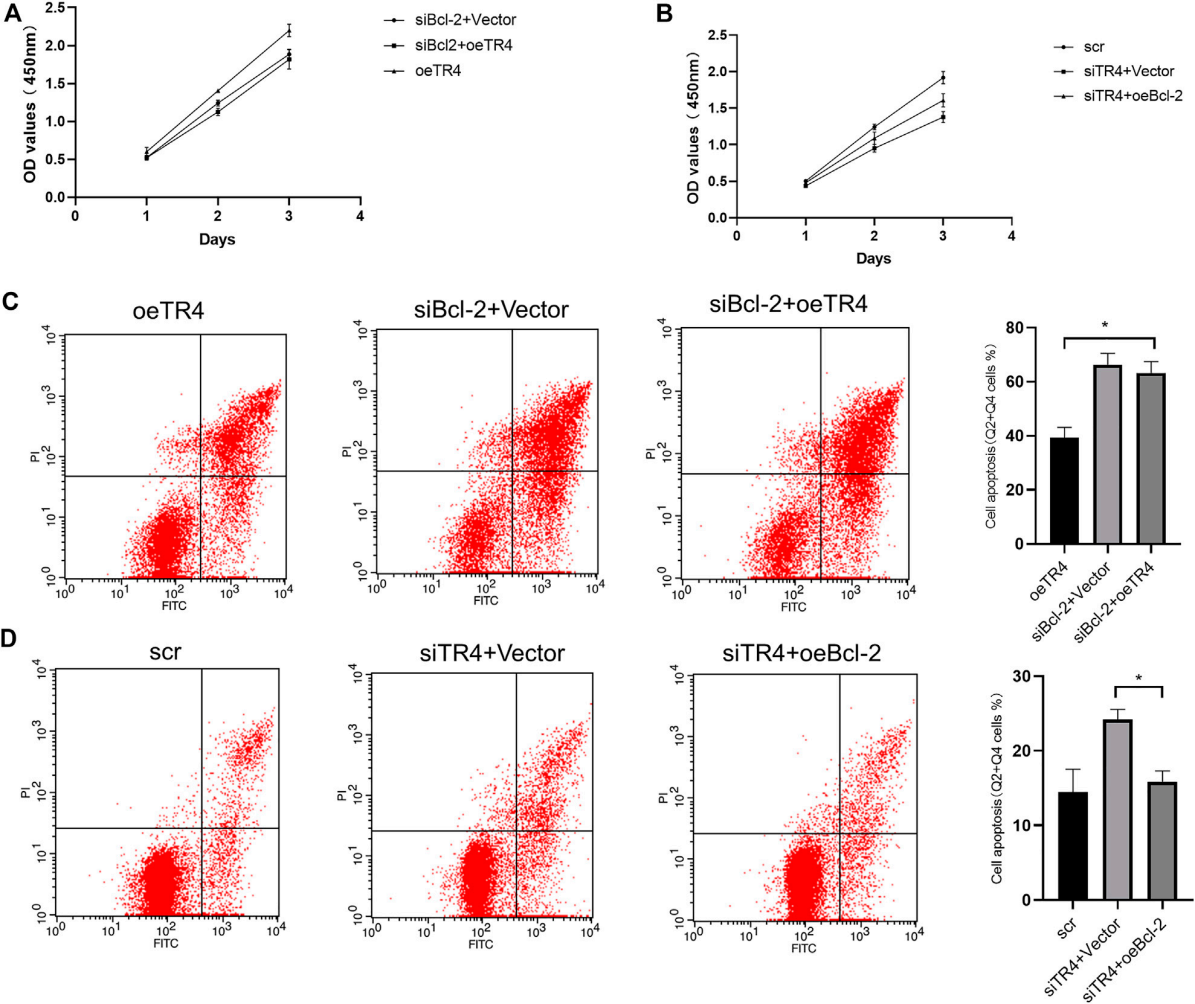
The effect of TR4 on apoptosis is dependent on Bcl-2 in T24 cells: **(A)** CCK8 was used to measure the proliferation after knocking down the Bcl-2 expression in oeTR4-T24 cells. **(B)** CCK8 was used to measure the proliferation after overexpressing Bcl-2 in siTR4-T24 cells. **(C)** Flow cytometry assay was used to evaluate the apoptosis after knocking down the Bcl-2 expression in oeTR4-T24 cells, *p < 0.05. **(D)** Flow cytometry assay was used to evaluate the apoptosis after overexpressing Bcl-2 in siTR4-T24 cells, *p < 0.05.

**FIGURE 6:**
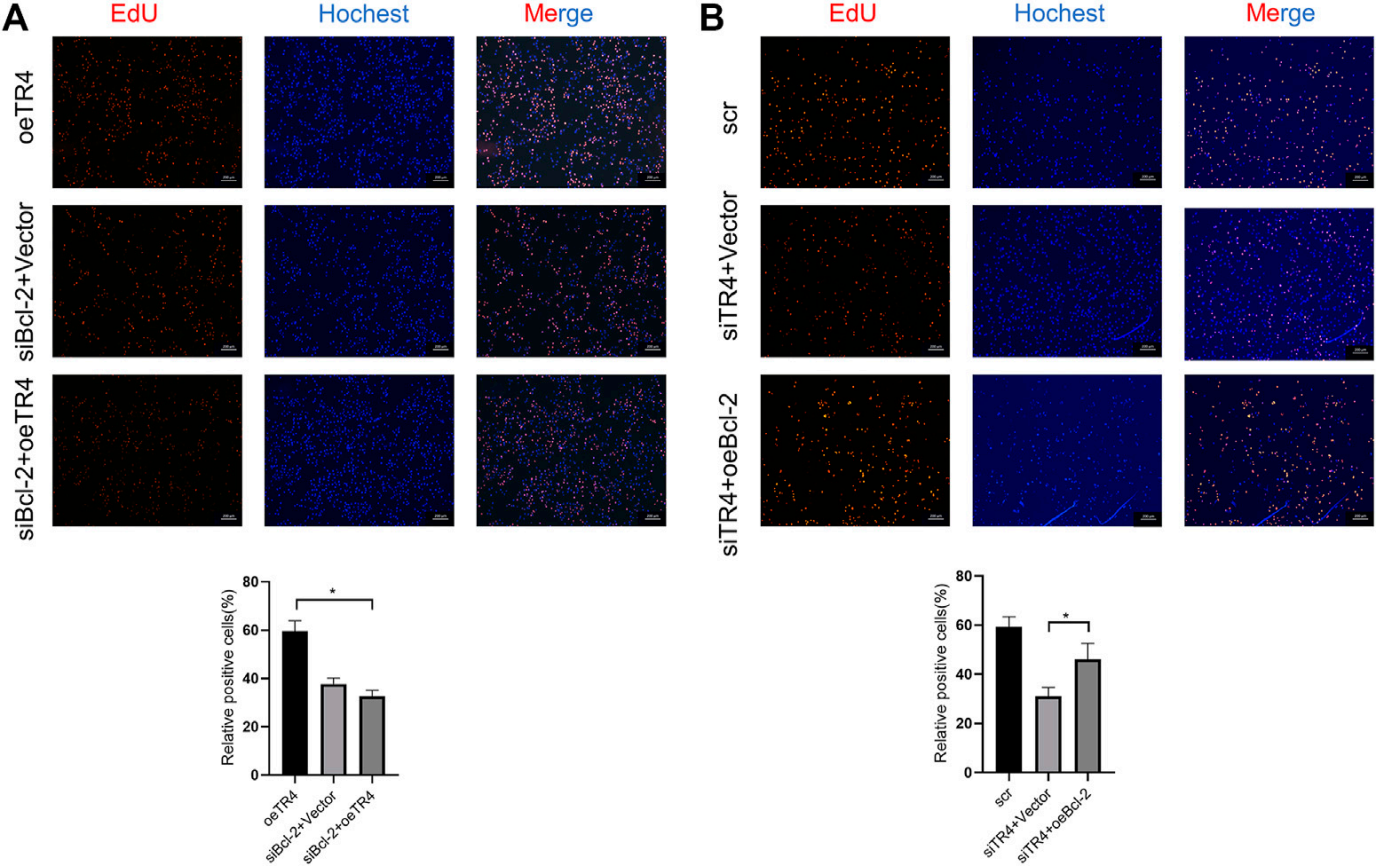
The effect of TR4 on viability is dependent on Bcl-2 in T24 cells. **(A)** EdU assay was used to evaluate the proliferation ability after knocking down the Bcl-2 expression in oeTR4-T24 cells, *p < 0.05. **(B)** EdU assay was used to evaluate the proliferation ability after overexpressing Bcl-2 in siTR4-T24 cells, *p < 0.05.

The knockdown of Bcl-2 in overexpressing TR4 bladder cancer cells abolished the proliferation (Figure 5A, Figure 6A, Supplementary Figures S2A, S3A). In TR4 silencing cells, Bcl-2 overexpression partly rescued the proliferation ability (Figure 5B, Figure 6B, Supplementary Figures S2B, S3B). In overexpressing TR4 cells, Bcl-2 knockdown increased the percentage of apoptotic cells (Figure 5C andSupplementary Figure S2C). In TR4 silencing cells, overexpression of Bcl-2 reduced the proportion of apoptosis cells (Figure 5D and Supplementary Figure S2D). These results demonstrate that TR4 promotes cell proliferation by suppressing apoptosis.

In summary, these results proved that TR4 exerted antiapoptotic effects by regulating the Bcl-2 expression.

### TR4 Promotes Bladder Cancer Growth *in vivo*

**FIGURE 7:**
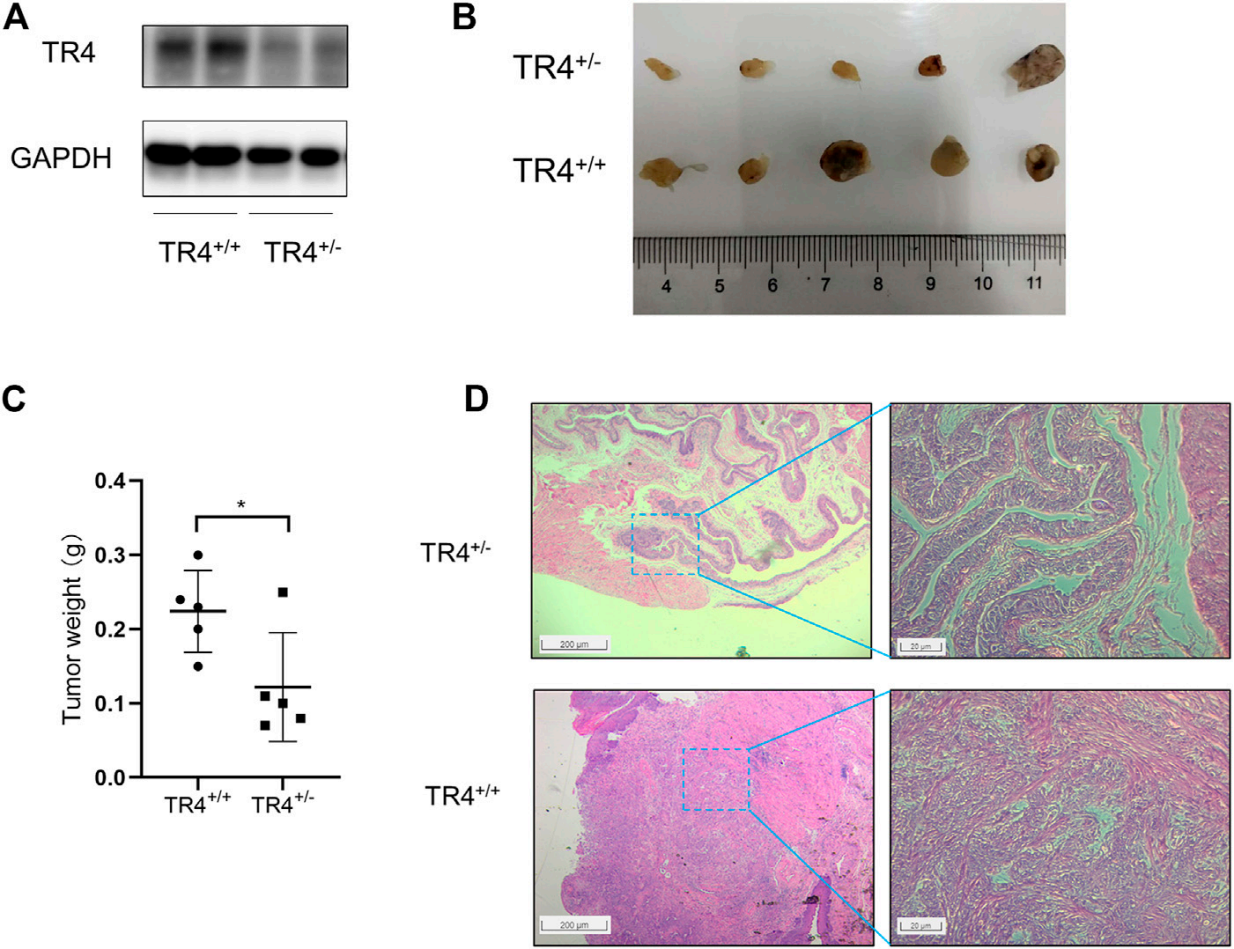
TR4 promotes BC growth *in vivo.* **(A)** TR4 expressions of TR4^+/+^ and TR4^+/−^ mice were confirmed by the Western blot. GAPDH was the internal reference. **(B)** The tumors were observed after 20 weeks in two different groups (TR4^+/+^ group and TR4^+/−^ group). **(C)** The tumors were weighed and analyzed with Student’s t-test between two groups, *p < 0.05. **(D)** Representative images of HE staining of tumors from the two groups. The left panel shows presentative images, while the quantification is shown in the right panel (magnification: ×100 (left) and 400× (right)).

Mice were divided into two groups: wild type (*n* = 5) and systemic TR4 knockdown mice (*n* = 5). The TR4 expression was confirmed by Western blotting (Figure 7A). To induce bladder cancer, we added 0.05% BBN to drinking water for the mice for 20 weeks. During the observation period of 20 weeks, we found that the tumor formation rate in the TR4^+/−^ group was higher than that in the control group (Figure 7B). More importantly, we found that knocking down the TR4 expression significantly retarded bladder tumor growth, which was consistent with the results *in vitro* (Figure 7B). In addition, the tumor volumes were significantly different between the two groups (Figure 7C). In addition, HE staining also indicated that the tumor was more aggressive in the control group than in the TR4^+/−^ group (Figure 7D). In summary, these results verify that TR4 knockdown dramatically inhibited the tumor growth *in vivo*.

## Discussion

In this study, we found that TR4 was upregulated in the bladder cancer tissues compared to the normal tissues. Moreover, we demonstrated that TR4 promotes bladder cancer progression by upregulating the Bcl-2 expression. These results indicate that TR4 plays an important role in the progression of bladder cancer.

In recent years, next-generation sequencing has identified many aberrant-expression genes or mutated genes in bladder cancer, which led us to understand bladder cancer initiation, development, and metastasis. The gene expression profiles indicated several distinct molecular signatures and subtypes in bladder cancer, providing us with a more accurate prediction of disease progression and a more useful therapy method for the disease. Previous studies reported that telomerase reverse transcriptase (TERT) mutations occurred in 70–80% of bladder cancers (Rachakonda et al., 2013; Leão et al., 2019). More evidence implied that TERT was associated with patient survival and disease recurrence (Rachakonda et al., 2013; Kurtis et al., 2016). Here, we found that TR4 was upregulated in bladder cancer at both mRNA and protein levels. Furthermore, TR4 mediated the proliferation of bladder cancer cells, suggesting that TR4 might function in bladder cancer.

TR4, as an orphan nuclear receptor without an identified ligand, has been proved to play an important role in tumorigenesis and tumor progression, including prostate cancer Lin et al. (2015b), renal cell carcinoma Bai et al. (2018), and hepatocellular carcinoma (Jin et al., 2018). Previous studies also revealed that TR4 played a cytoprotective role by altering the cell survival signaling (Lee et al., 2011; Liu et al., 2011). Therefore, many studies focused on its mediating effect on chemosensitivity among various cancers, including the prostate cancer Yang et al. (2013) and hepatocellular carcinoma (Shen et al., 2016). Shi group demonstrated that the TR4 expression was associated with the Bcl-2 expression (Zhu et al., 2016). In addition, TR4, as a transcriptional factor, might function by directly regulating the Bcl-2 expression (Kim et al., 2007). This study found that TR4 mediated bladder cancer cell proliferation *via* antiapoptosis. Our further study revealed that TR4 could bind to the Bcl-2 promoter region to increase the Bcl-2 expression. Our rescue experiments also showed that overexpression of Bcl-2 partly rescued the proliferation ability, suggesting that Bcl-2 was an important downstream factor in bladder cancer prognosis.

Interestingly, previous studies demonstrated that TR4 had the opposite effect on tumor initiation and progression (Lin et al., 2015b). In prostate cancer, TR4 acted as a tumor suppressor to prevent the tumor initiation by maintaining the DNA integrity (Lin et al., 2014). In contrast, TR4 was demonstrated to promote prostate cancer metastasis *via* CCL2 and EZH2 signaling. This study found that TR4 promoted the bladder cancer carcinogenesis in mice induced by 0.05% BBN. Consistent with the *in vitro* study, the tumor volume was significantly smaller and less aggressive in TR4^+/−^ mice than in the wild type mice. These evidences indicate TR4 plays an important role in the bladder cancer initiation.

In summary, these results demonstrate that TR4 has a positive role in promoting bladder cancer initiation and proliferation in *in vitro* and *in vivo* studies. These results may provide us with a new biomarker and a potential treatment target in the future.

## Data Availability

The original contributions presented in the study are included in the article/Supplementary Material; further inquiries can be directed to the corresponding authors.
