# Supplementary material for: Testicular Nuclear Receptor 4 Regulates Proliferation and Apoptosis of Bladder Cancer via Bcl-2
Source: Front Mol Biosci. 2021 Sep 20;8:670409. doi: 10.3389/fmolb.2021.670409 (PMC8488086; doi:10.3389/fmolb.2021.670409)
Supplement: Supplementary file 1 [file DataSheet1.docx]

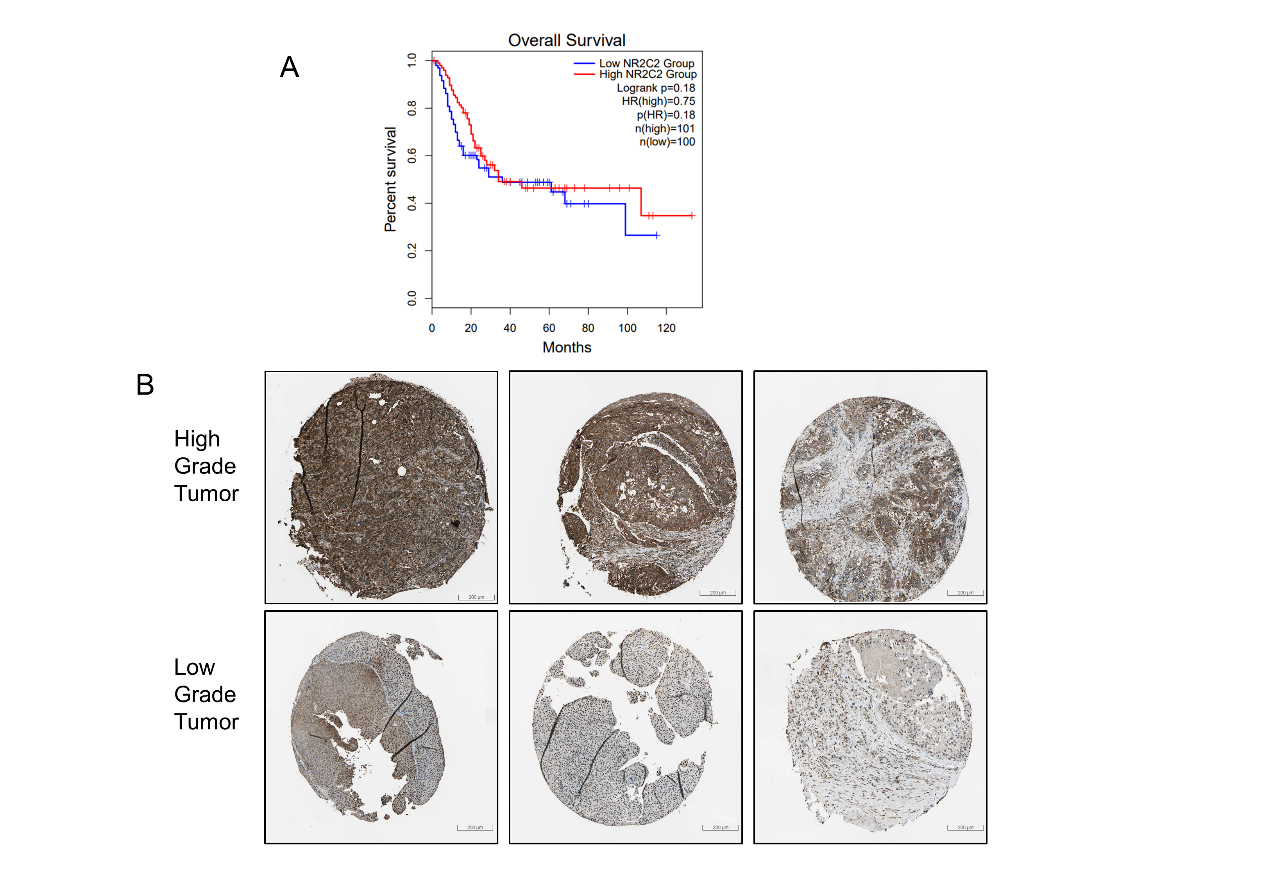


**Fig S1: The correlation between TR4 and bladder cancer.** (A) The relationship between overall survival and the expression of TR4 in bladder cancer. HR, hazard ratio. (B) IHC staining results of TR4 levels in high-grade bladder tumor and low-grade bladder tumor.


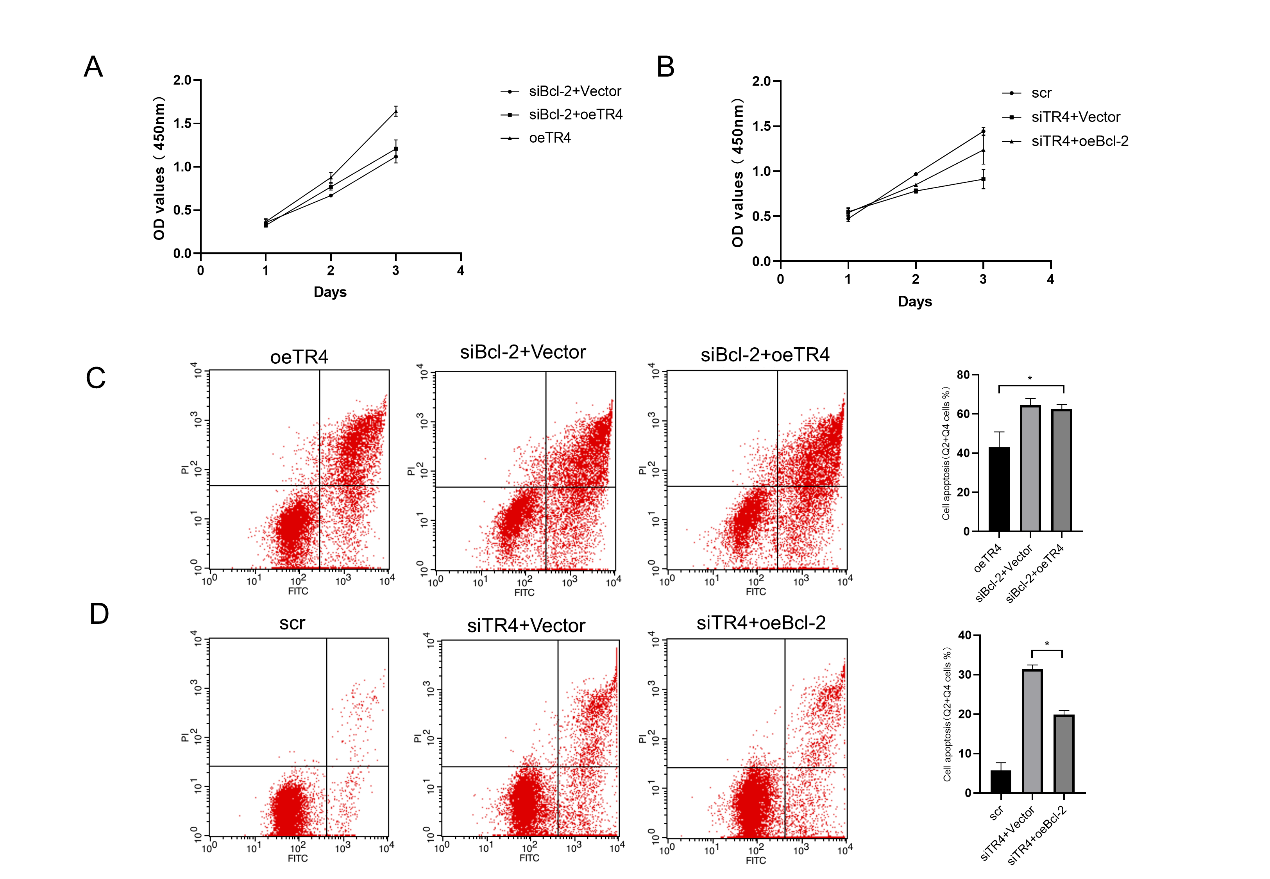


**Fig S2: The effect of TR4 on apoptosis can be reversed by Bcl-2 in UM-UC-3 cells.** (A) CCK8 was used to measure the proliferation after knocking down Bcl-2 expression in oeTR4-UMUC3 cells. (B) CCK8 was used to measure the proliferation after overexpressing Bcl-2 in siTR4-UMUC3 cells. (C) Flow cytometry assay was used to evaluate the apoptosis after knocking down Bcl-2 expression in oeTR4-UMUC3 cells; *p<0.05. (D) Flow cytometry assay was used to evaluate the apoptosis after overexpressing Bcl-2 in siTR4-UMUC3 cells; *p<0.05.


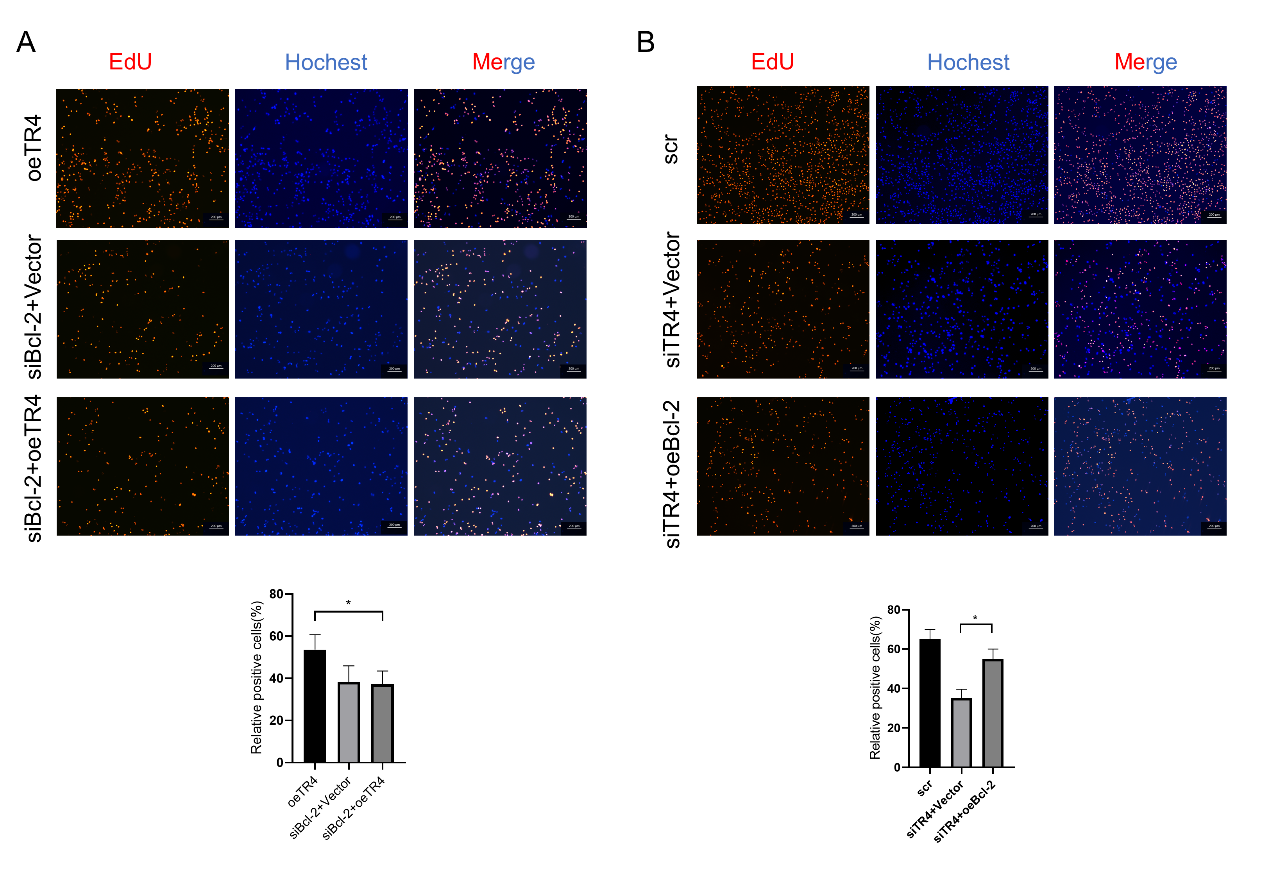


**Fig S3: The effect of TR4 on apoptosis can be reversed by Bcl-2 in UM-UC-3 cells.** (A) EdU assay was used to evaluate the proliferation ability after knocking down Bcl-2 expression in oeTR4-UMUC3 cells; *p<0.05. (B) EdU assay was used to evaluate the proliferation ability after overexpressing Bcl-2 in siTR4-UMUC3 cells; *p<0.05.
